# The Impact of Argon Plasma Coagulation in the Treatment of a Solitary Rectal Ulcer Syndrome Revealed by Massive Hemorrhage

**DOI:** 10.7759/cureus.23112

**Published:** 2022-03-13

**Authors:** Anass Nadi, Yasmine Cherouaqi, Zineb Oulammou, Hanane Delsa, Fedoua Rouibaa

**Affiliations:** 1 Gastroenterology and Hepatology, Mohammed VI University of Health Sciences (UM6SS), Casablanca, MAR

**Keywords:** colonoscopy, rectal pain, rectal bleeding, argon plasma coagulation, solitary rectal ulcer syndrome

## Abstract

Solitary rectal ulcer syndrome (SRUS) is a rare and chronic rectal condition that can result in a pelvic static disorder. Massive rectal bleeding is a rare manifestation of SRUS. The diagnosis is based on a combination of clinical, endoscopic, and histological findings. The management of bleeding ulcers is usually insufficient with the conventional treatment. Argon plasma coagulation (APC) has been reported to control bleeding. However, its role in healing and improving defecation symptoms is not unanimous in studies. Our case report features a 35-year-old male with terminal constipation and chronic rectal pain, taking laxatives and analgesics, who presented abundant rectal bleeding with hemodynamic instability. The colonoscopy showed two large bleeding rectal ulcers. The histological study of the biopsies was in favor of a solitary rectal ulcer. We have performed multiple sessions of APC. The bleeding was stopped after the first session and there was progressive healing and improvement of the rectal symptoms after other sessions. At 18 months follow-up, the patient is asymptomatic, and no longer uses analgesics and laxatives. Argon plasma coagulation is an effective treatment to control rectal ulcer bleedings. It also improves the healing process and clinical symptoms. However, further controlled studies are needed to support this hypothesis.

## Introduction

Solitary rectal ulcer syndrome (SRUS) is a benign, chronic and rare disorder. First described by Cruveilhier in 1828, its evolution is characterized by its inconstancy and its deep social impact [1]. The diagnosis is based on clinical, endoscopic, and histological criteria.

The clinical symptoms mainly include rectal dyschezia and minimal bleeding. Severe hemorrhage is unusual and dangerous [2]. Furthermore, there is, to this day, no agreement on the medical management of SRUS [3]. Argon plasma coagulation (APC) is a widely used hemostatic technique for upper gastrointestinal tract bleedings and arteriovenous malformations, however, for the rectal area, it is mainly used in the treatment of radiation proctopathy [3].

This study presents the case of a patient with SRUS revealed by massive rectal bleeding and treated with multiple APC sessions. The treatment’s goal was to stop the bleeding but also to evaluate the effectiveness of APC on the healing process and the clinical improvement of the patient.

## Case presentation

We present a case of a 35-year-old male admitted to the emergency room with massive rectal bleeding. The patient has a four-year history of terminal constipation with frequent use of digital manoeuvres, associated with chronic tenesmus and abdominal pain. He was treated with laxatives and analgesics. There was no history of underlying diseases and no surgical history.

In November 2019, the patient was admitted to the emergency room for massive rectal bleeding, slight abdominal pain, no transit disorders, and no vomiting. Upon admission, the examination found a conscient patient, hypotensive with a blood pressure of 90/55 mmHg, tachycardic at 108 beats/minute, normal respiratory rate at 18 breaths/minute, and pale skin. The abdominal examination was normal, the rectal examination showed no palpable mass and at withdrawal, the finger was smeared with blood. The rest of the examination was normal. The patient was admitted to an intensive care unit where he had a fluid resuscitation. The results of the first biological assessment are presented in Table 1.

**Table 1:** Laboratory finding MCHC: Mean corpuscular hemoglobin concentration, MCV: Mean corpuscular volume, WBC: White blood cells

	Laboratory value	Reference range
Hemoglobin	7.4 g/dl	13.0-18.0
Hematocrit	30%	39-53
MCHC	30 g/dl	31-36.5
MCV	80 fl	78-98
WBC	11000/mm³	4000-11000
Neutrophils	8500/mm³	1400-7700
Platelets	350000/mm³	150000-400000
Prothrombin	98%	70-100
Creatinine	8.2 mg/l	6.7-11.7
Urea	0.30 g/l	0.17-0.49

The patient was transfused with two units of blood. His hemodynamic status stabilized after three hours with a blood pressure of 110/80 mmHg, a heart rate of 68 beats per minute, a respiratory rate of 15 cycles per minute, and an oxygen saturation (SpO2) of 100%. But the rectal bleeding persisted. He was then administered a polyethylene glycol preparation using a split dosing protocol followed by an esophago-gastro-duodenoscopy and a colonoscopy.

**Figure 1:**
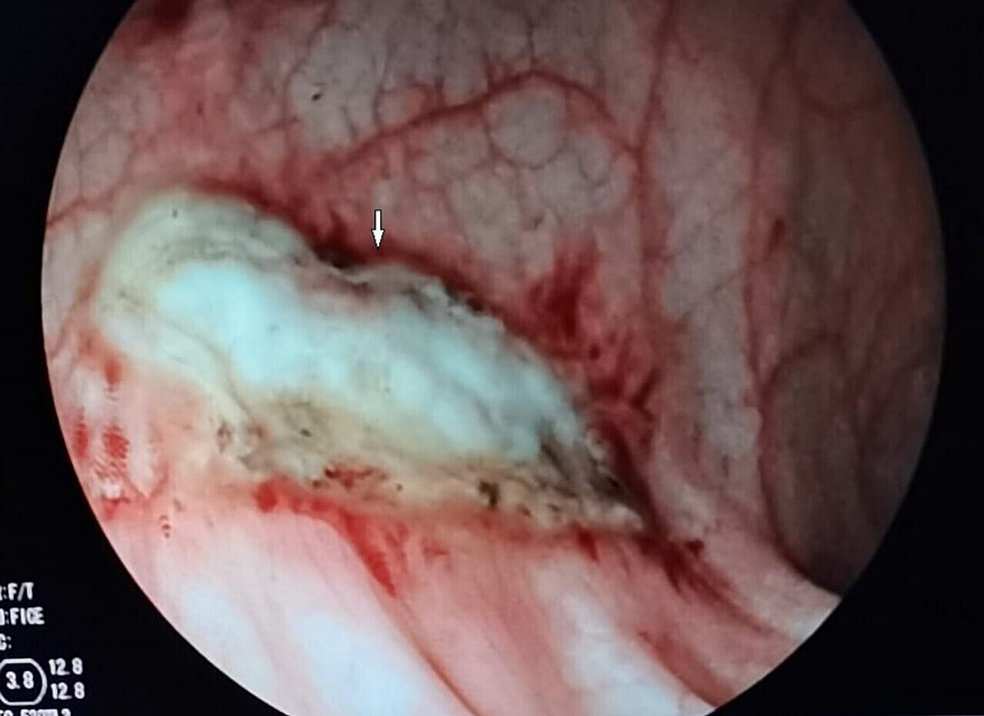
The first colonoscopy showing a large bleeding rectal ulcer (white arrows)

The upper endoscopy was normal, and the colonoscopy showed two large ulcers (Figure 1) that were contiguous and oval-shaped, respectively, and located at 14 cm and 16 cm of the anal margin while measuring 40 mm and 15 mm, respectively. The ulcers' margins were oedematous and bloody and the base of the ulcer was sclerotic and white. Biopsies of the margins were performed.

The endoscopic hemostasis was treated by APC (power: 45W, gas flow: 1.2l/min, device: maXium Beamer [KLS Martin, Freiberg, Germany]). The APC was aimed at both the ulcer margins and their base. The probe was not in contact with the mucosa. The patient’s evolution showed no more bleeding, and a clinical and biological improvement (control RBC count at 10g/dl, hematocrit at 40%).

The histological study of the biopsies was in favor of a solitary rectal ulcer with an architectural distortion of the crypts and fibromuscular and collagenous infiltration of the chorion. There were no histological signs of inflammatory bowel disease or neoplasm. Defecography showed no rectal prolapse.

**Figure 2:**
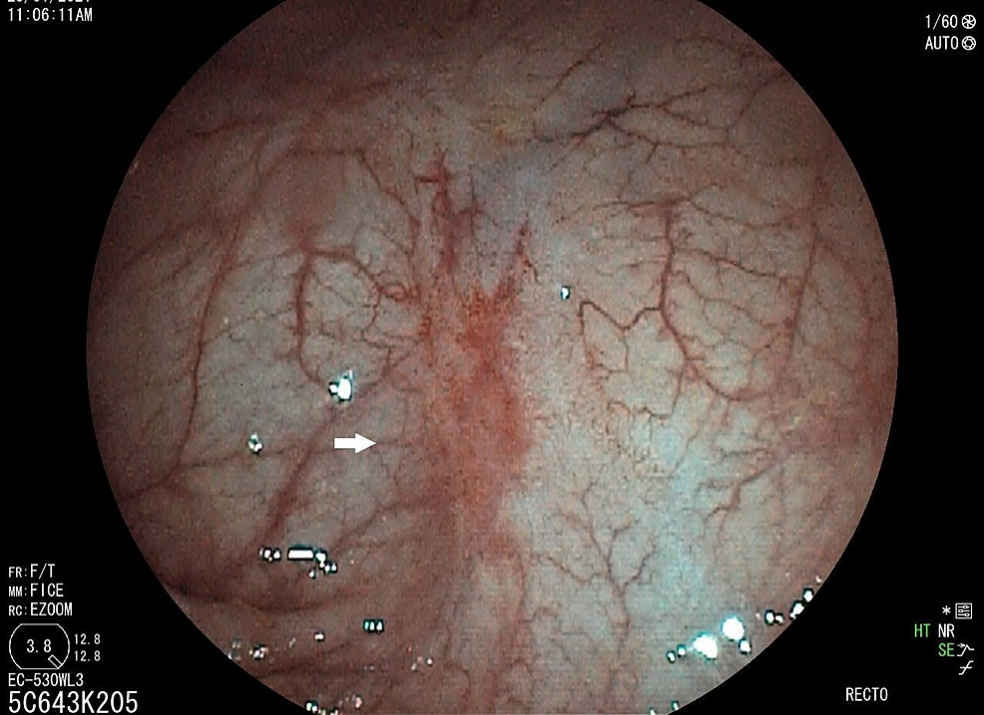
The second colonoscopy showing a progressive scarring aspect of the rectal ulcer with the persistence of erythematous areas (white arrows)

The follow-up colonoscopy at four weeks (Figure 2) showed the onset of ulcer healing, with the persistence of non-bleeding erythematous areas. Clinically, the patient no longer reported rectal bleeding, and with an improvement in terminal constipation, he presented less pushing effort, less anal pain, and less recourse to digital manoeuvres. We performed a second APC session at the edges and base of the ulcers (power: 40W; flow rate: 1l/min; device: maXium Beamer).

**Figure 3:**
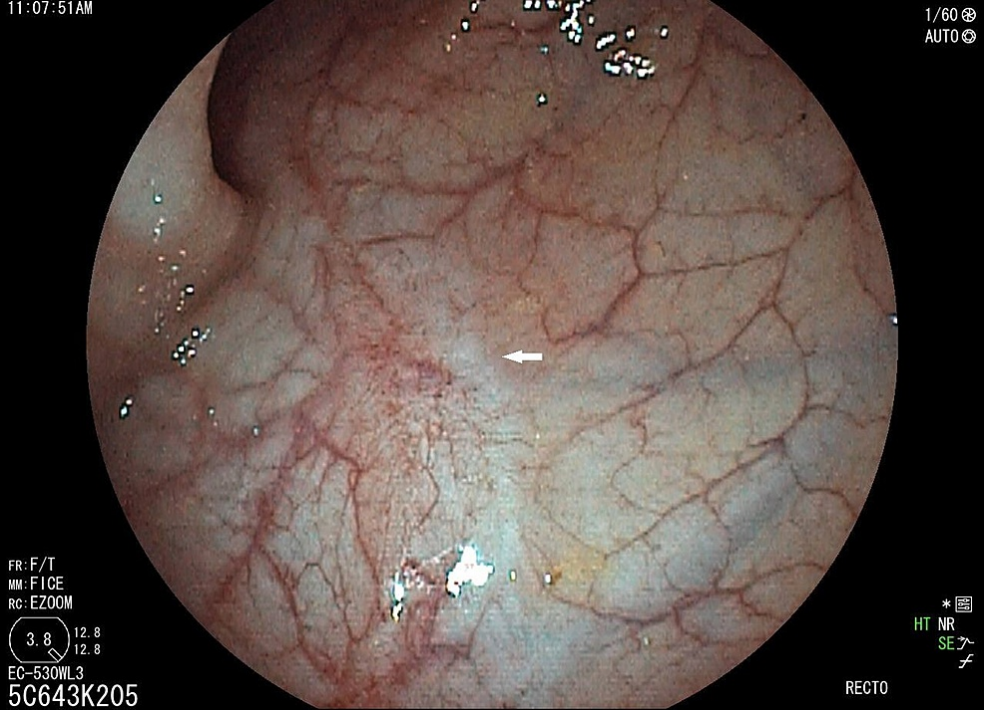
The third colonoscopy showing a reduction of the scarring and the size of the erythematous areas four weeks (white arrows)

The control colonoscopy (Figure 3) was performed four weeks after the second APC session, showed a significant reduction in the erythematous area which is traversed by a few bands of scarring. We carried out a third APC session (power: 40W; flow rate: 1l/min; device: maXium Beamer).

On the clinical level, we noticed an improvement in rectal dyschezia, as well as regular and less difficult defecation. The last control colonoscopy was done eight weeks after the third APC and showed complete scarring of the ulcers. Currently, with the follow-up at 18 months, the patient presents a regular transit without terminal constipation or rectal pain, recurrence of rectal bleeding, and has withdrawn from laxatives and analgesics.

## Discussion

Solitary rectal ulcer syndrome is a benign, chronic and rare rectal condition first described by Cruveilhier in 1829 [1]. It is characterized by a chronic ulcer of the rectal wall with the macroscopic and microscopic aspect of a chronic, traumatic and ischemic process. It is an underdiagnosed disorder with an incidence of 1/100000 and can affect both genders at any age with a slight predominance in men in their 40s and women in their 30s. A few cases in children and elderly people have also been reported [3].

The pathophysiology of SRUS is complex and multifactorial. It involves direct traumatic and local ischemic mechanisms. The mechanical evacuation techniques can also be responsible for the ulcer because of direct trauma [4]. In our case, the lesions were higher and couldn’t have been explained by digital manoeuvres. The clinical manifestations associated with the rectal syndrome include abdominal pain, tenesmus, dyschezia, and mucus discharge. Rectal bleeding is the most common symptom but is usually minimal and intermittent. Diarrhoea is described in 20% to 40% of cases [4]. For our patient, the SRUS was revealed by massive rectal bleeding engaging the prognosis of the patient. It is a rare manifestation of the syndrome [5].

Lower endoscopy is required for the diagnosis. An SRUS-typical lesion is present in 30% to 70 % of patients. The lesion is a white ulcer with a firm base and clean edges, circled by an erythematous mucosa, and is located in the anterior wall of the rectum usually between 3cm to 10cm from the anal margin. Its size varies between 0.5cm to 4 cm, usually between 1cm to 1.5 cm. However, this typical aspect is not consistent. Many other aspects have been described such as nodular or polypoid lesions. Rectal stenosis or lesions similar to the ones described in inflammatory bowel disease (IBD) or adenocarcinoma have been found [5]. The need for multiple biopsies is essential to eliminate other diagnoses such as adenocarcinoma or IBD. Our patient presented a characteristic histological aspect of SRUS and there was no histological finding in favor of IBD or neoplasm.

The treatment for SRUS is difficult and nonconsensual. To this day, there is no international recommendation on its management primarily due to the lack of evidence [6]. The choice of treatment depends on the severity of the symptoms and the existence of rectal prolapse. The main goals are the improvement of the symptoms and the quality of life, and ideally the scarring of the lesions, which is rarely achieved [6]. The management of SRUS includes medical treatment, perineal rehabilitation, endoscopic and surgical treatment.

The medical conservative treatment consists of transit regulation with the use of a high fiber diet and laxatives. The psychosocial factors must also be regulated. However, the level of evidence is low as only one study has assessed laxatives in the management of SRUS [7]. Sucralfate, 5-aminosalicylic acid (5-ASA), or corticosteroids rectal enemas have various efficiency according to a small series. Their long-time effect is not proven [8]. Biofeedback therapy is recommended to correct anorectal dysfunction. Surgery is recommended for patients presenting with complete rectal prolapse or not responding to the conservative treatment. Laparoscopic rectopexy with promontory fixation is currently the most used treatment [8].

As for the endoscopic treatment, APC is a newly proposed approach [6]. The technique is based on the use of a no-contact electrocoagulation device. Coagulation is done through a high-frequency monopolar current led by ionized argon gas. Some studies suggested a double effect of argon plasma. On one hand, it stops the bleeding and on the other hand, it could induce rapid scarring of the ulcerated lesions that would improve some of the symptoms such as dyschezia and rectal pain [3]. Only a few studies have compared the effect of APC on the healing of SRUS to the conventional treatment.

In a clinical case reported by Stoppino et al., a 64-year-old patient presented a large hemorrhaging solitary rectal ulcer with severe rectal pain. An APC was used following a four-session protocol with an interval of a month between each session. Thanks to the treatment, the bleeding stopped after the first session, the ulcer’s depth was reduced, the pain disappeared, and the patient no longer needed pain medication. The ulcer was almost completely healed after a nine-month follow-up [9]. In our case, we achieved the same results with three APC sessions after 18 months. The improvement of constipation in our patient can be explained by good healing of the rectal ulcer. Indeed, the APC-induced healing allowed an improvement of the rectal pain and also a reintegration of the rectal mucosa which allowed a reduction of a possible internal rectal prolapse. It is the same result as the one obtained by Stoppino et al. [9].

Since the publication of Stoppino et al.'s case report, there have been only two randomized controlled studies comparing the effect of APC to the effect of SRUS’s conventional treatment. The first study was published by Somani et al. in 2010. It includes 24 patients randomized into two groups, one undergoing APC and medical treatment (laxatives, high fiber diet, biofeedback, behavioral modification), and the other using medical treatment only. The results showed a high 66.7% healing rate for the group undergoing APC with a 16.7% healing rate for the other group. However, there was no significant pain improvement difference between the two groups (83.3% in the APC group for 66.7% for the other group, p=1) [8]

The second randomized study was published by Zergani et al. in 2017. It had the same goal as the Somani et al. study using 99 patients. The APC associated with conventional treatment was more efficient than the conventional treatment used alone with a response rate of 76% and 29%. As for pain improvement, the results weren’t statistically significant (83.5% in the APC group versus 62% for the group that didn’t use APC, p=0.36) [3].

In another prospective observational study, 14 patients who were not responsive to the conventional treatment (high fiber diet, rectal enemas, and biofeedback) were treated with several APC sessions. The bleeding was stopped in 100% of the cases and healing was achieved in 71% of cases (10 patients) after one to five sessions. No complication was observed three months after the last APC session [10]. This disease’s rarity would explain the difficulty of prospective and randomized studies.

## Conclusions

Solitary rectal ulcer syndrome is a benign, chronic and rare rectal disease, Its etiology remains unproven even if a combination of mucosal prolapse, trauma, and ischemia caused by excessive straining of stools may be important factors. The clinical symptoms mainly include rectal dyschezia and minimal bleeding. Severe hemorrhage is unusual and dangerous.

Our case report shows the positive impact of APC in the treatment of solitary rectal ulcer syndrome revealed by the massive lowering of bleeding. It is a promising technique with a therapeutic effect on bleeding, healing, and in the improvement of chronic clinical symptoms. Several APC sessions are necessary to achieve these therapeutic results. However, other studies are necessary to elaborate a consensual therapeutic protocol.
